# Evolution of voice after transoral laser cordectomy for precancerous lesions and early glottic cancer

**DOI:** 10.1007/s00405-021-06751-3

**Published:** 2021-03-18

**Authors:** Lucia Staníková, Karol Zeleník, Martin Formánek, Jana Seko, Radana Walderová, Peter Kántor, Pavel Komínek

**Affiliations:** 1 Department of Otorhinolaryngology and Head and Neck Surgery, University Hospital Ostrava, 17. Listopadu 1790, 708 52 Ostrava, Czech Republic; 2 Department of Craniofacial Surgery, Faculty of Medicine, University of Ostrava, Ostrava, Czech Republic

**Keywords:** Precancerous laryngeal lesions, Early glottic cancer, Cordectomy, Quality of voice, Vocal function

## Abstract

**Purpose:**

To evaluate voice quality evolution after a transoral laser cordectomy (TLC) for precancerous lesions and early glottic cancer.

**Methods:**

This prospective study enrolled 18 patients scheduled for TLC for high-grade dysplasia, Tis, T1, and T2 glottic squamous cell cancers, from May 2017 to March 2020. Patients were grouped according to the extent of TLC: Group I (*n* = 11, 61.1%): unilateral subepithelial or subligamental cordectomy; Group II (*n* = 7, 38.9%): unilateral transmuscular, total, or extended cordectomy. Voice quality parameters, including dysphonia grade (G), roughness (R), breathiness (B), maximal phonation time (MPT), jitter, and shimmer, were evaluated before, and at 6 weeks and 6 months after the TLC.

**Results:**

In Group I, the degree of G and R items remained without substantial improvement 6 weeks after surgery; however, improved above the pre-surgery level up to 6 months after surgery. The MPT, jitter, and shimmer did not change significantly at 6 weeks or 6 months post-TLC. In Group II, G, R, and B remained significantly impaired even 6 months post-surgery. Jitter, and shimmer worsened at 6 weeks, but reached preoperative levels at 6 months post-surgery. MPT was significantly worse at 6 weeks and remained deteriorated at 6 months post-surgery. All measured parameters were significantly worse in Group II than in Group I at 6 weeks and 6 months post-surgery. No patient required a phonosurgical procedure.

**Conclusion:**

After a TLC, voice quality evolution depended on the extent of surgery. It did not improve at 6 weeks post-surgery. Improvements in less extent cordectomies occurred between 6 weeks and 6 months post-surgery. Understanding voice development over time is important for counseling patients when considering phonosurgical procedures.

## Introduction

Transoral laser cordectomy (TLC) and radiotherapy are both considered highly effective treatment modalities for patients with precancerous lesions of the vocal cords and early glottic cancer [1, 2]. Both methods provide excellent disease-free intervals, overall survival, and larynx preservation for T1–T2 carcinoma [3–6]. However, TLC offers several advantages over radiotherapy, such as one-session therapy, short hospitalization, reduced morbidity, and high cost-effectiveness [2, 3, 7]. In addition to survival, voice outcomes are important for posttreatment quality of life; therefore, starting from the time TLC was introduced, voice outcomes have been frequently discussed [8]. According to several studies, long-term voice outcomes (6–24 months) have been excellent after a subepithelial or subligamental cordectomy, but after more extended surgeries, voice outcomes remain poor, similar to preoperative tumor-associated dysphonia [4, 8–10]. Based on those findings, TLC is currently recommended, even for voice professionals (e.g., singers, sports casters, public speakers etc.), when a subepithelial or subligamental cordectomy is planned.

To date, the time period for voice recovery after TLC has not been examined. It is highly important for voice professionals to know when their voice will return. Therefore, the present study aimed to evaluate the development of voice quality after an endoscopic cordectomy for precancerous lesions or early glottic cancer, during the early postoperative period, and to assess differences in voice pattern evolution with different extents of surgery.

## Materials and methods

This prospective study was performed in accordance with the Declaration of Helsinki, the requirements of good clinical practice, and all applicable regulatory requirements. It was approved by the Institutional Review Board. Written informed consent was obtained from all participants before any procedure was initiated.

### Patient selection, allocation, and follow-up

This study enrolled patients with high grade dysplasia, carcinoma in situ (Tis), T1, and T2 glottic squamous cell cancer that were scheduled for TLC, from May 2017 to March 2020. No patient received previous radiotherapy or surgical treatment. Patients with symptoms of gastroesophageal reflux were not included in the study.

Patients were divided into two groups, according to the extent of cordectomy. Group I included patients that underwent unilateral subepithelial or subligamental cordectomies (European Laryngology Society [ELS] type I and II resections). Group II included patients that underwent unilateral transmuscular, total, or extended cordectomies (ELS III, IV, and V resections) [11]. We evaluated voice quality before the cordectomy and at 6 weeks and 6 months after the surgical treatment.

### Evaluation of voice quality

#### Perceptual evaluation of voice quality

For perceived voice quality evaluation, voice recordings of patients were randomly analyzed by one of three specialists experienced in voice evaluation, based on the dysphonia grade (G), roughness (R), breathiness (B), items of GRBAS scale, which was introduced by Hirano in 1981. In this scale, the dysphonia grade is the perceived grade of hoarseness, including all voice components; roughness is an instability in voice intensity and frequency; breathiness is perceived air leakage on phonation [12]. Each parameter was scaled as follows: 0—normal, without perceived impairment; 1—small impairment; 2—moderate impairment; and 3—severe impairment. Voice recordings were made during the preoperative examination, 6 weeks and 6 months after TLC with lingWAVES software (version 2.5, Wevosys, Forchheim, Germany). During the recording, a microphone (Sound Level Meter Datalogger CENTER322, New Taipei City, Taiwan) was set at a constant distance of 30 cm from the patient’s mouth, and the patient named the months in a year with a normal voice.

#### Aerodynamic evaluation

The maximum phonation time (MPT, measured in seconds) was used to assess voice aerodynamics. The MPT was estimated with lingWAVES software (version 2.5, Wevosys, Forchheim, Germany). The patients recorded three trials of speaking the /a:/ vowel with maximum prolongation at a spontaneous, comfortable intensity, loudness, and pitch, after a maximal inspiration. The longest MPT was selected for further assessment.

#### Acoustic evaluation

The acoustic evaluation was performed with the Vospector program (provided with lingWAVES software). The patients recorded, in standard phonation, the /a:/ vowel at a comfortable frequency and intensity. We analyzed the 2 s after the middle part of the phonation. We estimated the percent jitter (reflecting short-term instability in voice frequency) and the percent shimmer (reflecting changes in voice amplitude) for each patient.

### Oncological follow-up

Each patient in our study was re-evaluated at 1 year after the cordectomy. We detected no locoregional persistence or recurrence of laryngeal carcinoma, based on narrow band imaging coupled with high definition endoscopy in a conventional examination [13].

### Statistical analysis

The evolution of voice parameters over time was ascertained and analyzed in each group separately. Thereafter, the differences between groups were analyzed. For G, R, and B items which are categorical ordinal data, comparison of absolute and relative frequencies was used for analysis. Numerical parameters of other parameters (MPT, jitter, shimmer) are expressed as the median and interquartile range (IQR), as well as the mean and standard deviation (SD). The Wilcoxon signed-rank test was used to evaluate changes in parameters between examinations. The groups were compared with the Mann–Whitney test. The significance level was set to 0.05. All analyses were performed with the R software (R version 4.0.2, http://www.r-project.org).

## Results

### Study group

This prospective study enrolled 25 patients. Of these, seven (28%) were excluded due to noncompliance with follow-up visits. Among the 18 remaining patients, 17 (94.4%) were men, 1 (5.6%) was a woman, and the median age was 67.0 years (range: 48–83). Group I included 11/18 (61.1%) patients with a mean age of 67.0 years. Group II included 7/11 (38.9%) patients with a mean age of 70.0 years. Age was not significantly different between groups.

### Evolution of voice quality

**Fig. 1:**
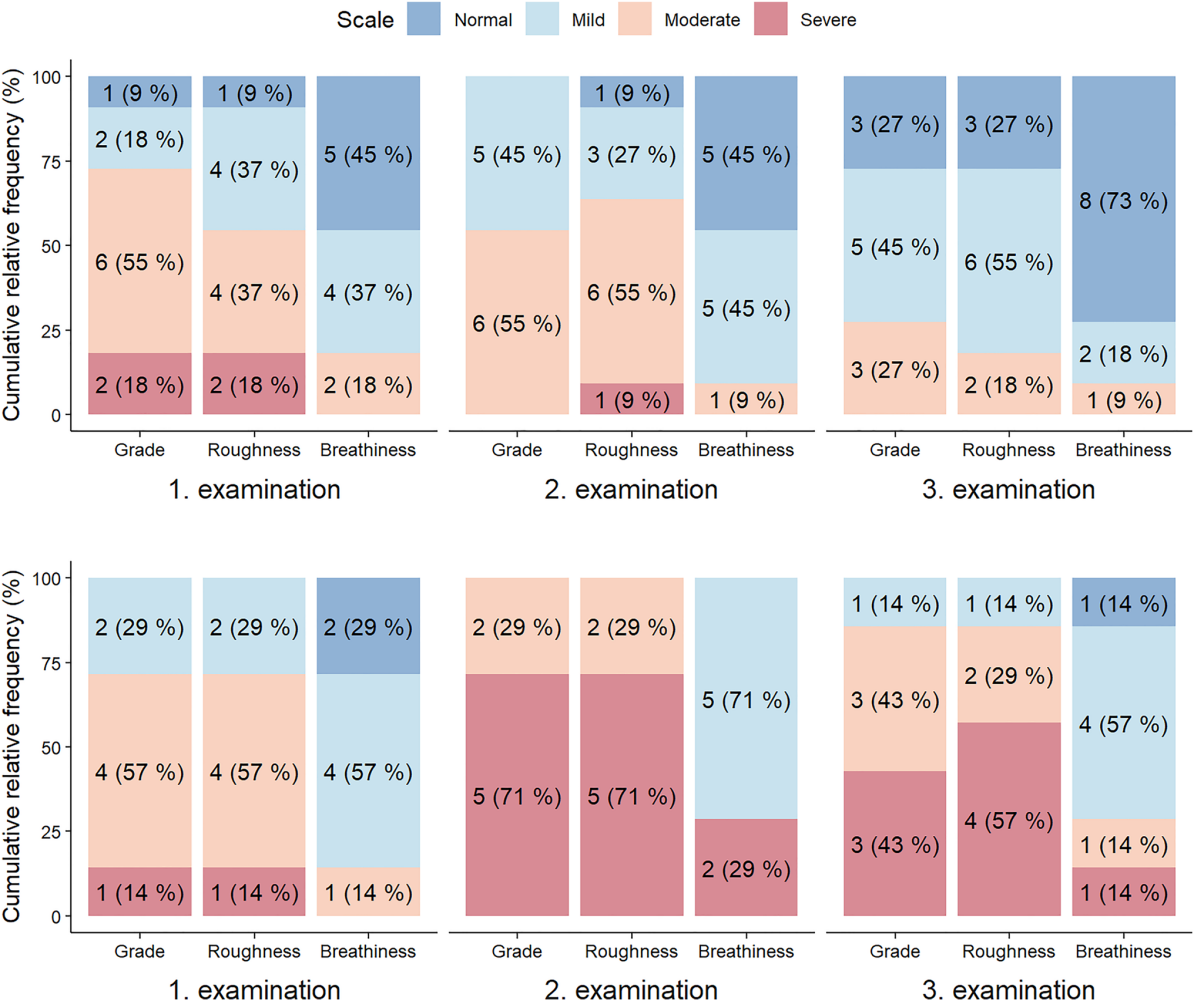
Evolution of grade, roughness and breathiness in time for Group 1 (upper part) and Group 2 (lower part). 1. Examination—preoperative; 2. examination—6 weeks after surgery; 3. examination—6 months after surgery

**Fig. 2:**
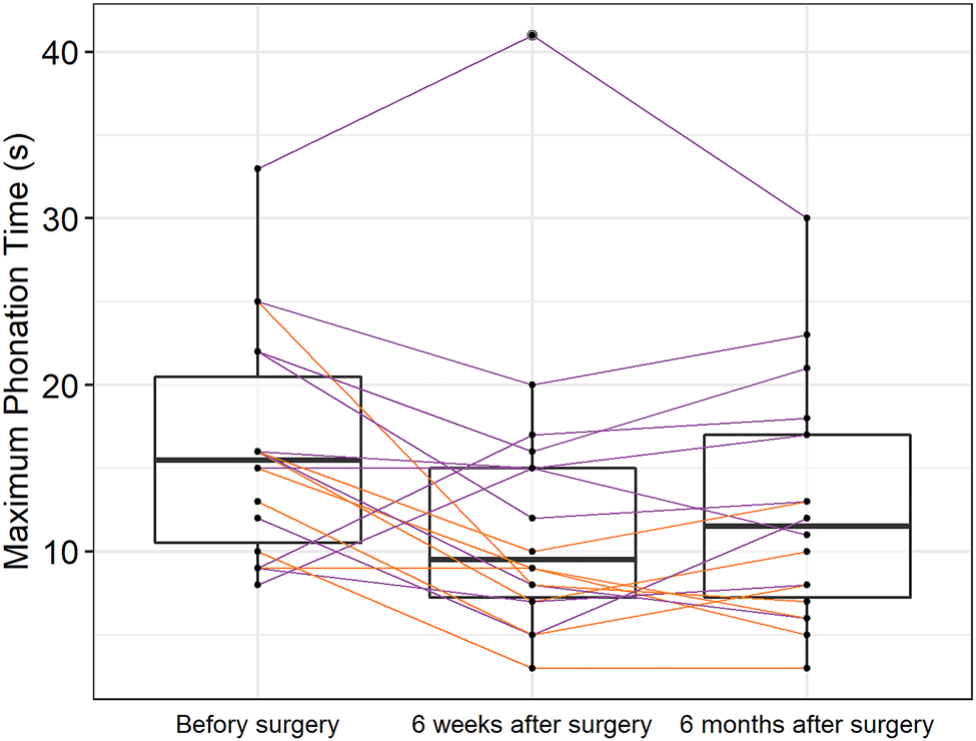
Changes in the maximum phonation time. Group 1: purple lines; Group 2: orange lines; boxplot shows the median (horizontal line), interquartile range (box), and the maximum and minimum scores

Evolution of perceived voice quality in time using G, R and B evaluation is showed in detail in Fig. 1. In Group I, the degree of G and R items remained without improvement to mild or normal value in more than half of patients 6 weeks after surgery. However, up to 6 months after surgery, G and R improved in most patients and were above the pre-surgery level. Item B did not worse in 6 weeks nor in 6 months after surgery (Fig. 1). Moreover, Group I showed no significant change in MPT over time, and jitter and shimmer did not change significantly between the preoperative examination and 6 weeks after surgery. However, at 6 months after surgery, jitter and shimmer improved significantly. Nevertheless, the preoperative and 6-month examinations did not differ significantly (Tables 1, 2; Fig. 2).

**Table 1:** Comparison of the changes in maximum phonation time (MPT), jitter and shimmer over time between Group 1 and Group 2

Examination	Group 1		Group 2		*P*^c^
	MED (IQR)^a^	Mean (SD)^b^	MED (IQR)^a^	Mean (SD)^b^	
MPT					
1	16.0 (10.5–22.0)	17.0 (7.8)	15.0 (11.5–16.0)	14.9 (5.3)	0.784
2	15.0 (10.0–16.5)	15.6 (9.6)	8.0 (6.0–9.0)	7.3 (2.5)	0.026
3	17.0 (11.5–19.5)	16.0 (7.0)	7.0 (5.5–9.0)	7.4 (3.3)	0.010
Jitter					
1	2.2 (0.9–6.6)	4.1 (4.5)	5.3 (2.4–9.8)	6.4 (5.0)	0.285
2	3.0 (0.5–4.8)	3.3 (3.1)	13.3 (10.3–16.2)	13.0 (5.0)	< 0.001
3	0.5 (0.3–2.1)	2.1 (3.4)	7.8 (4.9–11.2)	8.4 (6.0)	0.009
Shimmer					
1	19.6 (13.8–25.3)	21.9 (11.0)	22.5 (15.3–29.4)	24.0 (10.5)	0.375
2	19.2 (15.5–26.2)	20.4 (5.6)	32.9 (28.1–41.9)	34.7 (8.9)	0.004
3	13.9 (11.8–20.6)	15.5 (5.3)	25.5 (21.3–32.5)	27.0 (10.7)	0.020

*Group 1* European Laryngology Society type I and II resections, *Group 2* European Laryngology Society type III, IV and V resections, *1* preoperative, *2* 6 weeks post-surgery, *3* 6 months post-surgery

^a^The median and the interquartile range

^b^The mean and the standard deviation

^c^P value of the Mann–Whitney test

**Table 2:** Evolution of maximum phonation time, jitter and shimmer between examinations in Group 1 and Group 2

Examination	Group 1			Group 2		
	MED (IQR)^a^	Mean (SD)^b^	*P*^c^	MED (IQR)^a^	Mean (SD)^b^	*P*^c^
MPT progress						
1–2	− 2.0 (− 6.5; 3.5)	− 1.5 (6.6)	0.574	− 7.0 (− 8.5; − 6.0)	− 7.6 (5.1)	0.036
2–3	1.0 (− 0.5; 2.5)	0.5 (4.8)	0.448	0.0 (− 2.0; 3.0)	0.1 (3.0)	> 0.999
1–3	− 1.0 (− 3.5; 0.5)	− 1.0 (6.0)	0.356	− 6.0 (− 8.5; − 4.0)	− 7.4 (5.3)	0.022
Jitter progress						
1–2	− 0.4 (− 3.9; 0.5)	− 0.8 (4.9)	0.520	6.3 (4.5; 8.0)	6.5 (3.2)	0.016
2–3	− 0.5 (− 2.2; − 0.1)	− 1.2 (1.7)	0.019	− 4.8 (− 6.7; − 2.1)	− 4.6 (2.8)	0.016
1–3	− 1.9 (− 5.5; − 0.1)	− 2.0 (6.1)	0.147	1.4 (0.0; 3.9)	1.9 (3.3)	0.219
Shimmer progress						
1–2	0.7 (− 6.7; 4.7)	− 1.6 (9.6)	0.831	10.9 (8.7; 14.9)	10.7 (6.5)	0.031
2–3	− 4.7 (− 6.8; − 3.0)	− 4.9 (2.4)	< 0.001	− 7.4 (− 10.8; − 3.3)	− 7.7 (5.1)	0.016
1–3	− 1.3 (− 13.8; 0.7)	− 6.4 (11.3)	0.147	2.2 (− 2.1; 8.5)	3.0 (6.0)	0.375

*Group 1* European Laryngology Society type I and II resections, *Group 2* European Laryngology Society type III, IV and V resections, *1* preoperative, *2* 6 weeks post-surgery, *3* 6 months post-surgery

^a^The median and the interquartile range

^b^The mean and the standard deviation

^c^P value of the Wilcoxon signed-rank test

In Group II, all items (G, R and B) were significantly worse 6 weeks after surgery. Moreover, only partial improvement could be observed 6 months after surgery. In most patients, G and R items were scored as moderate to severe impairment even 6 months after surgery (Fig. 1). Group II showed a significant worsening of the MPT between the preoperative and 6-week examinations, and it remained significantly worse at 6 months post-surgery. Jitter and shimmer significantly worsened at 6 weeks after surgery, but they both improved at 6 months after surgery; however, the 6-month values were not significantly different from preoperative values (Tables 1, 2; Fig. 2).

### Comparison of voice quality evolution between groups

A comparison of the two groups revealed no preoperative differences in the measured parameters. However, in Group II, all measured parameters (G, R, B, MPT, jitter, and shimmer) were significantly worse than those in Group I at 6 weeks after surgery. Moreover, all parameters in Group II remained significantly worse than those in Group I, even at 6 months after surgery. (Table 1; Figs. 1, 2).

## Discussion

TLC and radiation therapy are the main treatment modalities for patients with early glottic cancer (Tis, T1–T2). Success is most frequently measured in terms of the local control rate, laryngeal preservation, or in the long term, overall and disease-free survival [3, 9, 14–18]. Endoscopic laser microsurgery costs significantly less than external beam radiotherapy; therefore, microsurgery has been advocated for the treatment of early glottic carcinomas, particularly T1a carcinoma [3, 7, 16, 19]. Nevertheless, radiotherapy is often considered the treatment of choice, due to the superior voice quality results [20], despite the comparable cure, similar larynx preservation rates, and lower cost of transoral laser microsurgery. Indisputably, aggravated voice quality significantly affects quality of life, particularly for voice professionals, which currently includes a wide range of professions.

Previous studies that compared transoral excisions and radiotherapy reported equivalent [2, 14, 21, 22] or better voice quality results with radiotherapy [23, 24]. However, a later study found that the superiority claim resulted from the heterogeneity of surgical procedures for T1 vocal cord cancer, and that different extents of cordectomy might be necessary for a single TNM classification. Therefore, it was suggested that it might be more reasonable to rate voice outcomes according to the extent of cordectomy, rather than the TNM classification [25, 26].

A typical example of voice outcome assessments, according to the TNM classification, rather than the extent of cordectomy, was the first randomized controlled trial conducted by Aaltonen et al., in 1998–2008 [27]. They compared voice results between TLC and radiation therapy at 2 years after the treatments for T1a vocal cord cancer. They found that breathiness and asthenia were significantly worse in patients after TLC than after radiotherapy. However, the overall voice quality did not differ significantly between groups at 2 years after treatment. Nevertheless, although the self-reported voice quality did not differ between groups, hoarseness had less of an impact on daily living activities for patients in the radiation group than for patients in the TLC group. When interpreting the results of that study, it is important to note that those authors did not report the exact extent of the surgical procedures; in particular, they did not specify the types of cordectomy performed. They only stated, that “tumor tissue was removed down to a macroscopically healthy muscle layer.” Therefore, it is likely that the transmuscular type of cordectomy was performed in that study. In that case, the results of the study should not be taken as representative of T1a cancers treated with a subepithelial or subligamental cordectomy. Same approach to evaluate voice after cordectomies was utilized by other authors [7, 14, 24].

Fink et al. chose a different approach to assessing voice outcomes in their retrospective study [25]. Those authors analyzed voice results in patients that underwent an ELS Type I, II, or III cordectomy. They determined that the Voice Handicap Index (VHI) improved or showed a trend of improvement postoperatively, and a perceptual analysis did not reveal any significant deterioration in voice quality. The shortcomings of that study were that the exact time point of the postoperative examination was not stated, and the voice parameters were ascertained retrospectively. The VHI was assessed between 1 and 12 months after surgery (median 7 months), and the perceptual analysis was performed between 1 and 3 months after surgery (median 1.9 months). Therefore, it was not possible to trace the evolution of voice quality during the postoperative period.

Currently, most surgeons agree that measuring the extent of cordectomy is crucial in evaluating voice outcomes after a TLC [10, 25, 26, 28]. We chose this approach in the present study. We divided the patients into two groups, according to the extent of cordectomy. Group I included unilateral subepithelial or subligamental cordectomies (ELS I and II cordectomies), and Group II included unilateral transmuscular, total, or extended cordectomies (ELS III, IV, and V resections). In addition to comparing the static differences between the groups, we compared the evolution of voice quality between groups, during the 6-week and 6-month periods post-surgery. Indeed, no previous study has published information about short-term voice quality after different extents of cordectomy. This information is important for voice professionals and for counselling patients about their plans to return to work.

Our 6-month data were consistent with data reported by Roh et al., who evaluated voice quality at 1 year after a TLC for T1 vocal cord cancer. Those authors reported considerable differences in both the subjective and objective voice outcomes that depended on the extent of surgery. ELS types I and II cordectomies led to significant voice improvements, but more advanced resections led to significantly worse voice outcomes that markedly influenced the quality of life and social activities [9]. Likewise, Peretti et al. showed significant voice improvements after ELS types I and II cordectomies; in those cases, the voice attained nearly normal parameters. On the other hand, after ELS types III, IV, and V cordectomies, the vocal outcomes at 6 months after surgery were not significantly different from the preoperative voice quality. Therefore, those authors concluded that ELS type I and II resections, when indicated, were adequate procedures, even for voice professionals [10]. Nevertheless, those studies only reported results after 6 or 12 months post-surgery, respectively.

The present study provided additional information about the post-surgical evolution of voice quality over a short time period in both groups. We found that, at 6 weeks after an ELS I or II cordectomy (Group I), G and R items remained without improvement; however, improved substantially between 6 weeks and 6 months after surgery. Thus, we could conclude that voice professionals would be not able to return to work for at least 6 weeks after those types of surgery. Furthermore, improvements in the voice above preoperative levels could be expected between 3 and 6 months after surgery. We also found that the other parameters did not significantly change postoperatively, in Group I. In contrast, in Group II, the G, R, and B were significantly worse at 6 weeks after surgery, and the voice did not improve even at 6-month-follow-up. Additionally, the MPT in Group II worsened significantly between the preoperative examination and 6 weeks after surgery, and it remained significantly worse than the preoperative level, even after 6 months. Jitter and shimmer were also significantly worse than the preoperative levels at 6 weeks after surgery; however, at 6 months after surgery, they were not significantly worse than the preoperative (tumor associated) level. Therefore, we concluded that after an ELS III, IV, or V cordectomy, the voice would not improve and might even become worse. Therefore, radiotherapy might be preferable for patients that consider voice quality crucial.

This issue was studied with a different methodology by van Loon et al. Those authors presented long-term voice outcomes for patients treated for extended T1 and limited T2 glottic carcinoma. Their patients underwent unilateral transmuscular (ELS type III) or bilateral subligamental (ELS type II) resections. The results of that study suggested that the majority of patients could expect to have mild to very moderate dysphonia 1 year postoperatively, based on ratings by experienced listeners and patient self-assessments [28].

Patients with severe dysphonia that previously underwent a total or extended cordectomy (ELS types IV and V resections) could be recommended for laryngeal framework surgery, or medialization laryngoplasty. When medialization surgery is considered, it is necessary to allow a prudent lapse of time between the tumor excision and the phonosurgical procedure [29–31]. A minimum 6-month period between the cordectomy and framework surgery is enforced to allow the vocal cord to scar and form a fibrous “neocord”. Then, voice recovery can be evaluated, and the risk of operating on a patient with undiagnosed early recurrence can be avoided [30]. The reported need for medialization surgery after a total or extended cordectomy was 14.2% [30]. In the present study, after 6 months, all patients had acceptable voice outcomes for casual communication. Therefore, no patient required laryngeal framework surgery or a medialization laryngoplasty.

## Conclusion

Our study showed that the evolution of voice quality after a TLC depended on the extent of the resection. Precancerous lesions and early glottic cancers that required limited surgery without muscular infiltration (i.e., ELS types I and II cordectomies) showed good voice quality outcomes. Thus, TLC should be offered for these lesions, even when the patient is a voice professional. However, patients should be informed that voice quality improvements require more than 6 weeks, and good voice quality can only be presumably achieved in 3–6 months after surgery. In contrast, when a more extensive cordectomy is planned, the patient should be informed that voice deterioration is expected, and if voice quality is essential for the patient, radiotherapy should be recommended.
